# Alveolar Abscess

**Published:** 1868-08

**Authors:** W. H. Shadoan


THE AUTHOR OF REMARKS ON DENTAL TISSUES.

### ALVEOLAR ABSCESS.

BY DR. W. H. SHADOAN.

[Continued from page 282.]

### HOW THEY ARE PRODUCED.

It will be well to state that the same laws govern alveolar abscess that govern abscesses in general. The healthy action of a single cell, or a number, may be disturbed by a faulty, or diseased supply of nutritious matter, or by mechanical violence, wounding or entirely obliterating them, either of which will deprive the cell or cells of their pure pabulum or food, and death is the result. When periosteal inflammation takes place, whether it be at the apex of the root or any other point, and continues until suppuration ensues, the periosteum at that point thickens, and as the matter accumulates, the periosteum is distended, and begins to press against the walls of the alveolus, and the consequence is, that the walls at the point affording the least resistance, are absorbed until there is an opening formed for the escape of the matter.

"Let us take, for instance, a case of common phlegmonous abscess, and trace it from its origin to its culmination. The inception depends upon the poisoning of pabulum, or mucus mass, by the accumulation of innutritious or effete matter, which disturbs the nutrient action of one or more cells, by depriving them of their pure pabulum or healthy liquid food. These salts and gasses being held in solution, have a tendency to diffuse themselves in every direction throughout the free juices of the flesh, and this predominance of chemical affinities disturbs the equipois of the currental movement denominated vitality, and is destructive in the exact proportion of its predominance." The healthy parts are constantly making an effort to stay the further advance of the abscess and to some extent limits the size of the sac. The process of the formation is by breaking down cell after cell, until the whole surrounding structure is destroyed; this may be slight or extended, owing to the poisonous condition of the juices, and the state of the health of the patient. There seems to be no settled theory among authors, just how, or by what *modus operandi* abscesses are formed. Some writers assert that the sac is formed by the distention of the periosteum, while others say that it is formed by the hardening of coagulable lymph, which is effused at that point. If the latter be true, then it is certain that the periosteum is destroyed by a sort of chemico-vital process so far as the pressure of the sac extends; then if the theory of the formation of the sac, by the distention of the periosteum be true, that membrane is changed somewhat in character; the healthy periosteum being a white fibrinous substance capable of powerful resistance, &c., while the sac of an abscess is of a different character, inasmuch as it is much more highly organized, and is capable of greater resistance than healthy tissue.

Prof. Harris says that "whenever there is intense or severe inflammation at the root, or in the alveolus infusion of coagulable lymph takes place, which hardening attaches itself to the tooth, and ultimately a sac is formed. This, as suppuration takes place distends and presses against the alveolus through which an opening is formed for the escape of the matter."

The character of abscesses is exceedingly variable owing to the constitutional peculiarities and susceptibilities, the condition of the parts immediately concerned, and to the cause producing it. There are exceptions however to all rules, but this is the general rule. Persons of a scrofulous temperament are more liable, as before stated, and an abscess in persons of this temperament takes the chronic form almost immediately; while those of good constitutions will recover with little or no treatment at all. When a part capable of suppuration is subjected to inflammation of the required intensity some of the small vessels give way and blood is effused into the surrounding parts; simultaneously with this rupture, or nearly so, the arteries begin to throw out a peculiar plastic matter, called coagulable lymph, this is capable of becoming organized and being thrown around the diseased parts, and between them and those which are healthy, it forms a barrier to the infiltration of extravasated fluids.

"By some strange process to us altogether inscrutable, the walls of lymph become vascular, and capable of performing the vital functions of secretion and absorption and by them the pus is furnished. As this secretion proceeds the previous contents of the abscess including the effused blood, are gradually absorbed, and fresh pus is deposited in their stead, so that if the tumor be opened at an early stage the pus will be more or less mixed with blood; but if the opening be delayed the cavity will be found to contain only pure pus."*

*Bond's Dental Medicine.

### WHERE SITUATED.

The point of attack is usually at the apex of the roots, but not always. In the superior teeth, abscess attacks the incisors sometimes on the side, some distance below the point, and especially on the buccal surface, in the bicuspids there is little difference from the incisors and canine teeth. In the molars the point of attack is frequently in the bifurcations of the roots, sometimes occupying the entire space, if at the apex of the root, the palatine or lingual is most likely to be affected, or in other cases, the anterior buccal root. The same will apply to the inferior teeth as to the superior, except in the single-root teeth. They are rarely attacked except at the apex of the root. The inferior molars are attacked usually, at the point of the roots, but sometimes between them, at or near the bifurcation; as to which of the roots is most liable, there is really no difference. If the cause is from mechanical violence, it will be at the point where the greatest injury is produced. Abscesses in childrens' teeth are produced more frequently by mechanical violence, than, probably, any other cause, and in the four anterior superior teeth than any others. The third molars and especially the inferior are more liable to the ravages of this disease than any other class of teeth; they are liable to be attacked at all points; probably not in every tooth, but there is no point but is liable to attack at some time or other. The temporary teeth are more liable to the disease than the permanent, and should be more carefully treated, from the fact that the parts about are more susceptible to injury than the adult teeth and jaws. The superior incisors will be found more liable than the canine teeth, and the ten anterior inferior teeth less liable than any other class.

### POINTS OF ESCAPE.

There are, perhaps, as many points of escape of the pus of abscesses as there are different points of attack. As a general rule, the pus will find an opening through the most yielding part involved. If an incisor is affected, it is usually at the apex of the root; especially if it is an inferior tooth; but here we will remark that the six anterior inferior teeth, are rarely ever affected with abscess. Should such be the case it will be at the apex of the root. Not so with the superior incisors; they may be affected very often, and at various points. If abscess be produced from a dead or decaying nerve it will be at the apex of the root, and the opening will be at that point, but not always, though usually through the external wall of the alveolus. If the tooth thus affected be a central incisor, the opening may take a central or anterior course; in that event, when the pus reaches the suture, it will take its course along that suture, and find an escape at the posterior border of the hard palate. There are cases on record, where the pus passed over the floor of the nasal cavity, and was discharged at the soft palate. Such cases, however, are seldom met with. The cases most usually met with, are those that discharge through the anterior wall of the alveolar process, at the apex of the root. There is very little difference in points of attack, as well as of discharge, in any of the ten anterior teeth. They having single roots, except occasionally the first bicuspid which are sometimes double. If a molar of the superior maxilla be the seat of an abscess, it may be at the point of the roots, at the bifurcation, or on one side of the root; if at the apex of the palatine root, the pus will usually be discharged through the process at the point of the root, or it may traverse the alveolus for some distance before it is discharged; the most usual is at the point opposite the apex. Abscesses of the buccal roots discharge their contents through the outer wall and usually at the nearest point. If the anterior buccal root has an abscess at the apex, it is sometimes the case that the discharge is into the maxillary sinus. When this is the case the treatment is complicated. The discharge from a third molar may be an inch or two from the seat of the disease, owing to its situation. There have been cases where the discharge was on the angle of the jaw, or on the side of the neck, and one or two cases where the pus escaped on the back part of the shoulder. A case of this kind was described to me, by an old and experienced Dentist some time since. In the inferior third molars, the discharge may be on the inner side of the jaw, or at the lower edge, and is sometimes mistaken for scrofula, especially if the patient be of a scrofulous diathesis. The discharge from the first and second inferior molars is always or nearly so at the point of attack.

Before dismissing this part of the subject I will mention a few cases met with in my own practice:

*First case.*—A little boy about eight years of age—of a manifest scrofulous diathesis, was brought to my office for consultation. On examination I found large abscesses (or an abscess,) situated at the roots of the first and second left superior temporary molars, ulcerated at the roots and discharging the pus over the posterior angle of the malar bone, just under the canthus of the eye. I inserted the probang, and could distinctly feel the permanent teeth.

*Second case.*—A lady had an abscess at the apex of the root of the left superior lateral incisor, it discharged pus at the apex; in a few days she called to have it further treated as it seemed somewhat indolent. After giving it such treatment as I considered necessary she asked me to look at the opposite side of her mouth, where, to my astonishment, I found a lump as large as a hazel nut, which, on being opened, was found to contain pus; a further examination showed that the latter proceeded from the left side of the mouth. Here were two points of discharge from one abscess, one at the point of the root affected, and the other at a point between the right lateral and cuspidatus, a distance of at least one inch from the first. Another feature in this case is, that the whole face swelled, and beneath the right angle of the inferior jaw was swollen more than any other part. At one time I had fears that suppuration might possibly take place at that point. The cause of so much swelling and inflammation was, I think, the malarious condition of the system. I have failed to say that the external opening may be through the gum and into the mouth, or it may be through the cheek and skin, making its appearance on the face, and if it be a lower tooth the pus may be discharged through the jaw avoiding the mouth altogether. A very remarkable case of fistulous opening through the inferior maxillary is reported by Mr. Bell, and on account of the singularity of it, I give his report, believing that it will be of benefit to some who have not seen it. This had resulted from an abscess in the socket of a dens sapientiæ of the inferior maxilla. The discharge had been kept up for two years previous to the time that the case was submitted to Mr. Bell for treatment. "At this time a funnel-shaped depression existed in the skin, which could be seen to the depth of nearly three-quarters of an inch, and a small probe could be passed through it into the sac of the abscess underneath the root of the tooth. The abscess had now remained open for nearly two years, during the latter of which the parts had been in the state I have described them. I removed the tooth, and as I had anticipated, no further secretion of pus took place, but so perfectly had the communication been established, that when the gum healed, it left, by its contraction, a fistulous opening, through which a portion of any fluid received into the mouth passed readily to the outside of the cheek, and I could, with care, introduce a fine probe completely through the passage. So free, in fact, was the communication that some of the hairs of the whiskers, with which the external portion of the depression was filled, grew through the external opening and appeared in the mouth."

### PUS.

Pus, under all circumstances, is nearly the same, and all chemists give to it the same chemical constituents. Pus, of a good or healthy quality, is of a creamy appearance, of a yellowish white color, inodorous, and opaque. Alcohol and heat coagulate it. In an analysis, by Schuilque it was found to contain albumen and water, a particular extractive substance, and a small quantity of soda, phosphate of lime, and other salts. "Normal pus consists essentially of two distinct parts: pus corpuscles, or pus globules, and a colorless, aqueous fluid liquor puris, in which the corpuscles are suspended." A variety of globules or corpuscles are described by other chemists, but the above is sufficient for our purpose, hence we will not occupy space in describing them.

Unhealthy pus, the kind usually found in abscesses of an ichorus character, is very irritating to the parts effected, and as long as that condition exists the parts will remain in a diseased condition. It is of a thin dark appearance, and is irritating.

"Although it is true that such pus as is called healthy indicates a convalescent state of an ulcer or abscess, the inference to be drawn from its appearance attaches exclusively to the parts which secrete it, while it may herald the abatement of local inflammation, it may, at the same time, give clear evidence of a state of disease incompatible with integrity of organs, or with life itself. Suppuration of the eye, liver, or of the lungs would be a very serious matter, however healthy the pus might be." *

Some writers have considered suppuration a curative process, and have regarded the pus a valuable covering for the granulations or new growth of flesh, and so it is in some cases, but there are many exceptions.

Some parts of the body have a much greater disposition to form pus when inflamed than others. The cellular tissue, skin, and mucous membrane are very prone to suppurate, while the fibrous tissues manifest no disposition to it.

"Pus is modified, by the nature of the part where it is formed, by the constitution of the individual, by various accidents occuring in the process of its formation, and by cer-

* Macartney on Inflammation.

tain obscure laws which control the phenomena of these affections, which are called specific. It will also present different appearances, as it may be mixed with other fluids, as blood, saliva, bronchial mucus, etc." †

"When pus is irritating it is not so to the surface which secretes it, but to the adjoining healthy structure over which it flows.

"Pus is heavier than water, and this quality assists us in distinguishing it from mucus.

"Mr. Hunter is of opinion that it is coagulable in muriate of ammonia, which peculiarity distinguished it from mucus and all other natural secretions, but this test was disputed.

"From the fact that hard and inflammatory tumors, in the course of inflammation become soft and yielding, and filled with pus, it was naturally supposed that the original solid parts were converted into this fluid, it is now well ascertained that such is not the case, but that pus is secreted by the arteries." ‡

TO BE CONTINUED.

† Macartney on Inflammation.

‡ Bond's Dental Medicine.

